# Imp-L2, a putative homolog of vertebrate IGF-binding protein 7, counteracts insulin signaling in *Drosophila *and is essential for starvation resistance

**DOI:** 10.1186/jbiol72

**Published:** 2008-04-15

**Authors:** Basil Honegger, Milos Galic, Katja Köhler, Franz Wittwer, Walter Brogiolo, Ernst Hafen, Hugo Stocker

**Affiliations:** 1 Zoological Institute, University of Zürich, Winterthurerstrasse 190, CH-8057 Zürich, Switzerland; 2 Institute for Molecular Systems Biology (IMSB), ETH Zürich, Wolfgang-Pauli-Strasse 16, CH-8093 Zürich, Switzerland; 3 Current address: Chemical and Systems Biology, 318 Campus Drive, Clark Building W200, Stanford University Medical Center, Stanford, CA 94305-5174, USA

## Abstract

**Background:**

Insulin and insulin-like growth factors (IGFs) signal through a highly conserved pathway and control growth and metabolism in both vertebrates and invertebrates. In mammals, insulin-like growth factor binding proteins (IGFBPs) bind IGFs with high affinity and modulate their mitogenic, anti-apoptotic and metabolic actions, but no functional homologs have been identified in invertebrates so far.

**Results:**

Here, we show that the secreted Imaginal morphogenesis protein-Late 2 (Imp-L2) binds *Drosophila *insulin-like peptide 2 (Dilp2) and inhibits growth non-autonomously. Whereas over-expressing *Imp-L2 *strongly reduces size, loss of *Imp-L2 *function results in an increased body size. *Imp-L2 *is both necessary and sufficient to compensate Dilp2-induced hyperinsulinemia *in vivo*. Under starvation conditions, *Imp-L2 *is essential for proper dampening of insulin signaling and larval survival.

**Conclusion:**

Imp-L2, the first functionally characterized insulin-binding protein in invertebrates, serves as a nutritionally controlled suppressor of insulin-mediated growth in *Drosophila*. Given that Imp-L2 and the human tumor suppressor IGFBP-7 show sequence homology in their carboxy-terminal immunoglobulin-like domains, we suggest that their common precursor was an ancestral insulin-binding protein.

## Background

Insulin/insulin-like growth factor (IGF) signaling (termed IIS) is involved in the regulation of growth, metabolism, reproduction and longevity in mammals [1-3]. The activity of IIS is regulated at multiple levels, both extracellularly and intracellularly: the production and release of the ligands is regulated, and normally IGFs are also bound and transported by IGFBPs in extracellular cavities of vertebrates [4]. IGFBPs not only prolong the half-lives of IGFs, but they also modulate their availability and activity [5]. Besides the classical IGFBPs (IGFBP1-6), a related protein called IGFBP-7 (or IGFBP-rP1, Mac25, TAF, AGM or PSF) has been identified as an insulin-binding protein [6]. Although the reported binding of IGFBP-7 to insulin awaits confirmation [7,8], it can compete with insulin for binding to the insulin receptor (InR) and inhibit the autophosphorylation of InR [6]. Furthermore, IGFBP-7 is suspected to be a tumor suppressor in a variety of human organs, including breast, lung and colon [6,9-13]. A recent publication demonstrates that IGFBP-7 induces senescence and apoptosis in an autocrine/paracrine manner in human primary fibroblasts in response to an activated *BRAF *oncogene [14].

IIS is astonishingly well conserved in invertebrates. In *Drosophila*, IIS acts primarily to promote cellular growth, but it also affects metabolism, fertility and longevity [15,16]. Seven insulin-like peptides (Dilp1-7) homologous to vertebrate insulin and IGF-I have been identified as putative ligands of the *Drosophila *insulin receptor (dInR) [17]. These Dilps are expressed in a spatially and temporally controlled pattern, including expression in median neurosecretory cells (m-NSCs) of both brain hemispheres. The m-NSCs have axon terminals in the larval endocrine gland and on the aorta, where the Dilps are secreted into the hemolymph [17-19]. Ablation of the m-NSCs causes a developmental delay, growth retardation and elevated carbohydrate levels in the larval hemolymph [18,19], reminiscent of the phenotypes of starved or IIS-impaired flies.

The *Drosophila *genome does not encode an obvious homolog of the IGFBPs. Furthermore, genetic analyses of IIS in *Drosophila *and *Caenorhabditis elegans *have not revealed a functional insulin-binding protein so far. Here, we report the identification of the secreted protein Imp-L2 as a binding partner of Dilp2. *Imp-L2 *is not essential under standard conditions, but flies lacking *Imp-L2 *function are larger. Under adverse nutritional conditions, Imp-L2 is upregulated in the fat body and represses IIS activity in the entire organism, allowing the animal to endure periods of starvation.

## Results

### Genetic screen to identify negative regulators of IIS

**Figure 1:**
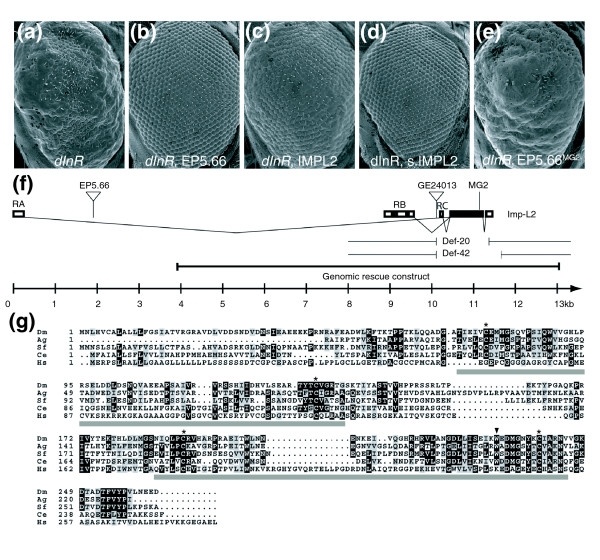
*Imp-L2 *overexpression suppresses *dInR*-induced growth. **(a-e) **Scanning electron micrographs of compound eyes. All flies (females) carry the GMR-*Gal4 *and UAS-*dInR*^*wt*^transgenes. The *dInr*-dependent big eye phenotype (a) is suppressed by EP5.66 (b). UAS*-Imp-L2 *(c) and the stronger UAS*-s.Imp-L2 *(d) also suppress, but EP5.66 driving the mutant *Imp-L2*^*MG*2 ^allele can no longer suppress the *dInR *overexpression phenotype (e). **(f) **Genomic organization of the *Imp-L2 *locus. The mutant alleles and P-element insertions used in this study are indicated. MG2 marks the point mutation in the EMS allele *Imp-L2*^*MG*2 ^that generates a premature stop codon. **(g) **Alignment of Imp-L2, its orthologs in invertebrates and the putative human ortholog IGFBP-7. Black and gray boxes indicate amino acid identity and similarity, respectively. The triangle marks the premature stop codon in Imp-L2^*MG*2^. Asterisks mark the cysteines forming the two disulfide bridges. The gray bars indicate the Ig domains. Dm, *Drosophila melanogaster *Imp-L2; Ag, *Anopheles gambiae *CP2953; Sf, *Spodoptera frugiperda *IBP; Ce, *Caenorhabditis elegans *zig-4; Hs, *Homo sapiens *IGFBP-7.

We reasoned that the overexpression of a Dilp-binding protein that impinges on the ligand-receptor interaction should counteract the effects of receptor overexpression. *dInR *overexpression during eye development (by means of a GMR-*Gal4 *strain, in which the Gal4 protein is overexpressed in photoreceptor neurons, and a UAS-*dInR*, which expresses dInR when activated by Gal4) results in hyperplasia of the eyes, a phenotype that is sensitive to the levels of the Dilps [17]. A collection of enhancer-promoter (EP) elements, which allow the overexpression of nearby genes (F.W., W.B., H.S., D. Nellen, K. Basler and E.H., unpublished work), was screened for suppressors of the *dInR*-induced hyperplasia (Figure 1a). A strong suppressor (EP5.66, Figure 1b) carried an EP element 8.5 kb upstream of the *Imp-L2 *coding sequence (Figure 1f). Two different UAS transgenes, both containing the *Imp-L2 *coding sequence but varying in strength, confirmed that the suppression was caused by *Imp-L2*. Whereas the weaker UAS-*Imp-L2 *(containing 5' sequences with three upstream open reading frames) only partially suppressed the *dInR*-induced overgrowth (Figure 1c), UAS-*strong.Imp-L2 *(UAS-*s.Imp-L2*, lacking the 5' sequences) completely reversed the phenotype (Figure 1d). In addition, a point mutation in the *Imp-L2 *coding sequence (see below) abolished the suppressive effect of EP5.66 (Figure 1e). Imp-L2 is therefore a potent antagonist of *dInR*-induced growth.

*Imp-L2 *has previously been shown to be upregulated 8–10 hours after ecdysone treatment [20,21]. It encodes a secreted member of the immunoglobulin (Ig) superfamily containing two Ig C2-like domains. Whereas several orthologs of Imp-L2 are present in invertebrates such as arthropods and nematodes, the homology in vertebrates is confined to the second Ig C2-like domain, which is homologous to the carboxyl terminus of human IGFBP-7 (Figure 1g). The carboxy-terminal part of IGFBP-7 differs considerably from the other IGFBPs, possibly accounting for the affinity of IGFBP-7 for insulin [6]. Interestingly, Imp-L2 has been shown to bind human insulin, IGF-I, IGF-II and proinsulin, and its homolog in the moth *Spodoptera frugiperda*, *Sf*-IBP, can inhibit insulin signaling through the insulin receptor [22].

### Overexpression of Imp-L2 impairs growth non-autonomously

**Figure 2:**
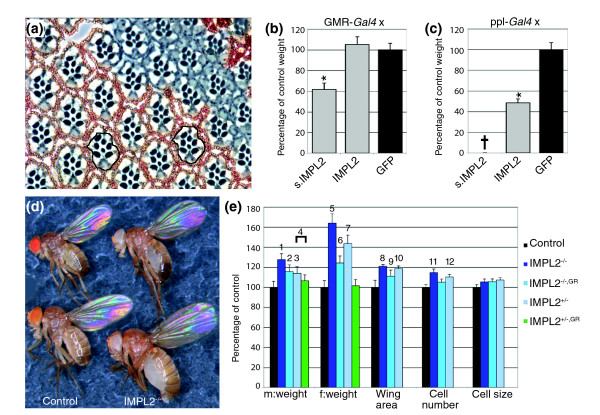
Imp-L2 controls body and organ size. **(a) **Tangential section through an adult eye containing an *Imp-L2 *overexpression clone marked by the lack of red pigment. Within the clone, the size of the ommatidia is reduced. Wild-type ommatidia close to the clone are also smaller (compare black circled areas). **(b) **Eye-specific overexpression of UAS*-s.Imp-L2 *reduces male body weight (-38.3%, *P *= 7 × 10^-42^). **(c) **Overexpression of UAS*-Imp-L2 *by ppl-*Gal4 *results in a 56.1% weight reduction in male flies, whereas ppl-*Gal4 *driven expression of UAS*-s.Imp-L2 *results in lethality (†). *P *= 3 × 10^-47^. **(d) **Loss of *Imp-L2 *function increases body size in males (top) and females (bottom). **(e) **Analyses of male and female weights. Wing area, cell number and cell size were assessed in female adult wings. GR indicates *Imp-L2 *genomic rescue construct. *P*-values are indicated by numbers as follows: 1, 2 × 10^-33^; 2, 8 × 10^-18^; 3, 9 × 10^-16^; 4, 6 × 10^-7^; 5, 3 × 10^-46^; 6, 1 × 10^-24^; 7, 8 × 10^-31^; 8, 2 × 10^-7^; 9, 4 × 10^-4^; 10, 4 × 10^-7^; 11, 3 × 10^-7^; 12, 1 × 10^-4^. Genotypes: 'control' *y*, *w*/*w*; 'Imp-L2^-/-^' *y, w*; *Imp-L2*^*Def*42^/*Imp-L2*^*Def*20^; 'Imp-L2^+/-^' for the weight analysis (e) *y, w*; *Imp-L2 *^*Def*20^/+; 'Imp-L2^+/-^' for the wing analysis (e) *y, w*; *Imp-L2 *^*Def*42^/+; 'Imp-L2^-/-^, GR' *y, w*; *Imp-L2 *^*Def*42^/*Imp-L2 *^*Def*20^, *GR-57*; 'Imp-L2^+/-^, GR' *y, w*; *Imp-L2 *^*Def*20^, *GR-57*/+. *P*-values were determined using unpaired Student's *t*-test against the control except in (4) where the weight of IMP-L2^+/-^ was compared to IMP-L2^+/-, GR^. n = 40 for the weight analysis in (b,c,e); n = 12 for the wing analysis in (e). Error bars represent s.d.

To further assess the function of Imp-L2 as a secreted inhibitor of insulin signaling, we ectopically expressed *Imp-L2 *using various Gal4 drivers. Strong ubiquitous over-expression of *Imp-L2 *by Act-*Gal4 *led to lethality with both UAS transgenes. Whereas driving UAS-*s.Imp-L2 *by the weaker ubiquitous arm-*Gal4 *driver also resulted in lethality, driving UAS-*Imp-L2 *generated flies that were decreased in size and weight (-15% in males and -29% in females, data not shown) but eclosed at the expected ratio and had wild-type appearance. By generating clones of cells that over-express *Imp-L2*, we confirmed that cell specification and patterning were normal in *Imp-L2*-overexpressing ommatidia (Figure 2a). However, a reduction of cell size was observed in the clones. This reduction seemed to be non-autonomous because wild-type ommatidia close to the clone were also reduced in size. Given the convex nature of the eye we were unable to quantify the effects of *Imp-L2 *overexpression on more distantly located ommatidia. Eye-specific overexpression of both UAS-*Imp-L2 *and UAS-*s.Imp-L2 *by GMR-*Gal4 *led to a strong reduction in eye size (data not shown). Whereas the GMR-*Gal4*, UAS-*Imp-L2 *flies were of normal size, body weight was reduced by 38.3% and development was delayed by one day in GMR-*Gal4*, UAS-*s.Imp-L2 *male flies (Figure 2b). Next, we used the ppl-*Gal4 *driver to over-express *Imp-L2 *in the fat body, a tissue that can be expected to produce and secrete Imp-L2 more efficiently than the eye. Driving UAS-*s.Imp-L2 *by ppl-*Gal4 *was lethal, whereas ppl-*Gal4*, UAS-*Imp-L2 *flies showed a pronounced reduction in body size (Figure 2c) and were delayed by 2 days. Both the size decrease and the developmental delay are characteristic phenotypes of reduced IIS such as in *chico *mutants [23], supporting the hypothesis that Imp-L2 acts as a secreted negative regulator of this pathway.

Next, we assessed the effect of *Imp-L2 *overexpression on phosphatidylinositol(3,4,5)trisphosphate (PIP_3_) levels using a green fluorescent protein-pleckstrin homology domain fusion protein (tGPH) that specifically binds PIP_3 _and serves as a reporter for PIP_3 _levels *in vivo *[24]. The amount of membrane-bound tGPH reflects signaling activity in the phosphoinositide 3-kinase/protein kinase B (PI 3-kinase/PKB) pathway. Overexpression of *dInR *resulted in a severe increase of membrane PIP_3 _levels (Additional data file 1, Figure S1A,B). Co-overexpression of *Imp-L2 *together with *dInR *reduced the PIP_3 _levels (Additional data file 1, Figure S1D), similar to the effect caused by PTEN (Additional data file 1, Figure S1C), a negative regulator of IIS. Therefore, Imp-L2 inhibits PI 3-kinase/PKB signaling upstream of PIP_3_, without affecting dInR levels (Additional data file 1, Figure S1B',D').

### Size increase in *Imp-L2 *mutants

We used two strategies to generate loss-of-function mutations in *Imp-L2*. First, we performed an ethylmethane-sulfonate (EMS) reversion screen in which we selected mutated chromosomes carrying EP5.66 that no longer suppressed the *dInR *overexpression phenotype (Figure 1a). One allele (*Imp-L2*^*MG*2^) containing a point mutation resulting in a premature stop at amino acid 232 was identified in this way (Figure 1e,f). This truncation destroys the conserved cysteine bridge of the second Ig domain (Figure 1g). Overexpression of the truncated *Imp-L2 *version had no inhibitory effect on size (Figure 1e), suggesting that *Imp-L2*^*MG*2 ^is a functional null allele.

Second, we generated additional *Imp-L2 *alleles by imprecise excision of GE24013 (GenExel), a P-element located 349 bp upstream of the ATG start codon of the *Imp-L2-RB *transcript (Figure 1f). We obtained *Imp-L2 *deletions (Def20, Def42) lacking the entire coding sequence. Heteroallelic combinations of the mutant alleles increased body size: whereas mutant males showed a 27% increase in body weight, mutant females were 64% heavier (Figure 2d,e). Introducing one copy of a genomic rescue construct (Figure 1f) [25] into homozygous mutant flies reverted the weight to the level of *Imp-L2*^+/- ^flies, which were already heavier (+14% in males, +44% in females, Figure 2e) than the controls. By measuring the cell density in the wing, the size increase could be attributed primarily to an increase in the number of cells, because cell size was only slightly affected (Figure 2e). Apart from the size increase, the flies lacking *Imp-L2 *appeared completely normal, eclosed with the expected frequency and were not delayed. Thus, under standard conditions, *Imp-L2 *loss-of-function dominantly increases growth by augmenting cell number without perturbing patterning, developmental timing or viability.

The weight difference was more pronounced in mutant females than in males, although the increases in wing area and cell number were similar (Figure 2e and data not shown). This differential effect was caused by enlarged ovaries in *Imp-L2 *mutant females (data not shown).

### Imp-L2 binds to and antagonizes Dilp2

**Figure 3:**
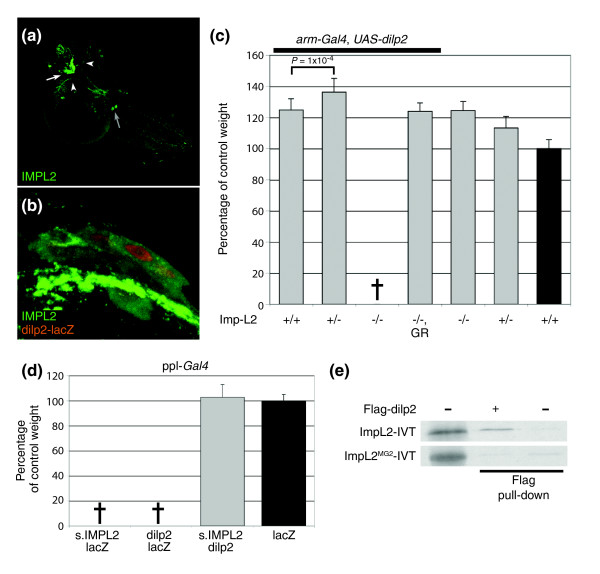
Imp-L2 binds Dilp2 and counteracts its activity. **(a,b) **Antibody staining of larval brains with an Imp-L2 antibody (green). (a) Specific neurons of both brain hemispheres, the subesophageal ganglion region (gray arrow) and the corpora cardiaca (white arrow) express Imp-L2 protein. The corpora allata are innervated by Imp-L2 expressing axons. White arrowheads mark the Dilp-producing m-NSCs. (b) In larvae carrying a dilp2-*lacZ.nls *transgene, co-staining with β-galactosidase and Imp-L2 antibodies reveals that the seven dilp-expressing m-NSCs also produce low levels of Imp-L2. **(c) **The size increase of arm-*Gal4*, UAS-*dilp2 *flies is dominantly enhanced by reducing *Imp-L2 *levels. In an *Imp-L2*^-/- ^background, *dilp2 *overexpression results in lethality, which can be rescued by a copy of the *Imp-L2 *genomic rescue construct (GR). **(d) **Overexpression of *dilp2 *as well as of *Imp-L2 *at high levels by ppl-*Gal4 *causes lethality, whereas concomitant overexpression of *dilp2 *and *Imp-L2 *yields flies of wild-type size. The lacZ transgene was introduced to rule out a dosage effect of the UAS/Gal4-system. **(e) **Imp-L2 binds Dilp2. *In-vitro*-translated, ^35^S-labeled wild-type (ImpL2-IVT, about 32 kDa) or mutant (ImpL2^MG2^-IVT, about 30 kDa) Imp-L2 (lane 1) was incubated with cell lysates of either non-transfected (lane 3) or stably transfected S2 cells expressing Flag-Dilp2 (lane 2). Imp-L2 could only be pulled down in the presence of Dilp2. The Imp-L2^MG2 ^mutation abolished Dilp2 binding. Genotypes in (c): 'Imp-L2^+/-^' *Imp-L2*^*Def*42^/+; 'Imp-L2^-/-^' *Imp-L2*^*Def*42^/*Imp-L2*^*Def*20^; 'Imp-L2^-/-^, GR' *Imp-L2*^*Def*42^/*Imp-L2*^*Def*20^, *GR-57*; 'control' (black bar) arm-*Gal4*, UAS-*GFP*. *P*-values were determined using unpaired Student's *t*-test (n = 40, except for bars 1–3 in (c): bar 1, n = 31; bars 2 and 3, n = 17). Error bars represent s.d.

The facts that Imp-L2 is a secreted protein and that removal of Imp-L2 function did not rescue either *chico *or *PI3K *mutant phenotypes (data not shown) are consistent with the hypothesis that Imp-L2 acts upstream of the intra-cellular IIS cascade at the level of the ligands. Immunohistochemistry in larval tissues revealed that, besides strong expression in corpora cardiaca (CC) cells (Figure 3a and Additional data file 1, Figure S2D), Imp-L2 protein was also weakly expressed in the seven m-NSCs that produce Dilp1, Dilp2, Dilp3 and Dilp5 (Figure 3b) and project their axons directly to the subesophageal ganglion, the CC, the aorta and the heart [19,26]. Thus, Imp-L2 potentially interacts with some of the Dilps directly at their source. We therefore tested for genetic interactions of *Imp-L2 *with the *dilp *genes. A deficiency (*Df(3L)AC1*) uncovering *dilp1-5 *not only dominantly suppressed the *dInR*-mediated big eye phenotype [17], but also dominantly enhanced the small eye phenotype caused by eye-specific overexpression of *Imp-L2 *(Additional data file 1, Figure S3). *dilp2 *is the most potent growth regulator of all *dilp *genes [18]. Weak ubiquitous overexpression of *dilp2 *by arm-*Gal4 *caused an increase in body and organ size [18], and this phenotype was dominantly enhanced by heterozygosity for *Imp-L2 *(Figure 3c). In homozygous *Imp-L2 *mutants, expression of *dilp2 *under the control of *arm*-Gal4 caused lethality, reminiscent of strong *dilp2 *expression [18]. Expressing *Imp-L2 *and *dilp2 *individually at high levels in the fat body also caused lethality, but coexpression resulted in viable flies of wild-type size (Figure 3d). Thus, *Imp-L2 *decreases the sensitivity to high insulin levels and is sufficient to rescue the lethality resulting from *dilp2*-induced hyperinsulinemia.

It has previously been shown that Imp-L2 can bind human insulin and insulin-related peptides [22]. To address whether Imp-L2 binds Dilp2, we constructed a Flag-tagged version of Dilp2, which is functional (data not shown). Using *in vitro *translated, ^35^S-labeled Imp-L2 together with Flag-Dilp2 extracted from stably transfected S2 cells, we could show that Imp-L2 binds Dilp2 *in vitro *(Figure 3e). A truncated form of Imp-L2 lacking a functional second Ig domain (like that produced by the *MG2 *allele) failed to bind Dilp2 (Figure 3e).

### Imp-L2 is essential under adverse nutritional conditions

**Figure 4:**
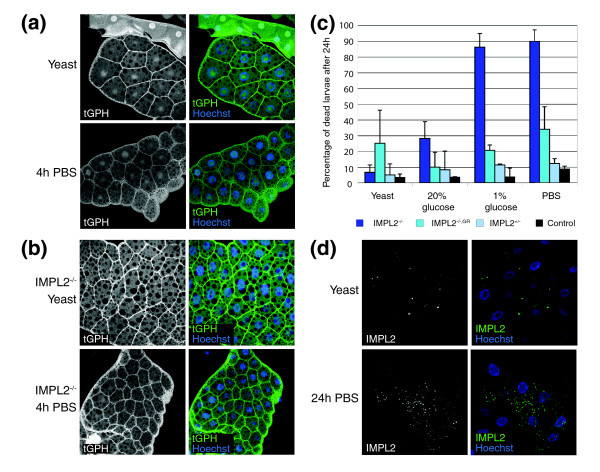
Imp-L2 is necessary for blocking *dInR *signaling under starvation. **(a,b) **tGPH fluorescence (green, showing PIP_3 _levels and thus indicating IIS activity) in the fat body of feeding third instar larvae under different nutritional conditions. Nuclear staining (Hoechst) is shown in blue in the right panels. (a) Under normal conditions ('yeast'), IIS activity is high in wild-type feeding third instar larvae. Upon starvation, only little PIP_3 _localizes to the membranes of fat body cells. (b) In *Imp-L2 *mutants, IIS activity is higher than in control larvae and only slightly reduced after 4 h PBS starvation. **(c) **Survival of *Imp-L2*^*Def*42^/*Imp-L2*^*Def*20^early third instar larvae is severely compromised under starvation conditions. One copy of the genomic rescue construct (GR) suffices to restore viability. Heterozygous larvae were *Imp-L2*^*Def*42^/+, control larvae *y, w*/*w*. Larvae (40) were subjected for 24 h to 20% glucose, 1% glucose or PBS. The experiment was repeated twice. **(d) **In starved larvae (*y, w*), Imp-L2 protein expression (green) is induced in fat body cells after 24 h PBS starvation. Imp-L2 is localized to vesicle-like structures but not detectable under normal nutritional conditions. Genotypes: (a,d) *y, w*; (b) *y, w*; *Imp-L2*^*Def*42^/*Imp-L2*^*Def*20^.

Despite being a potent inhibitor of Dilp2 action, Imp-L2 is not essential under standard conditions. Hyperactivation of the dInR pathway leads to increased accumulation of nutrients in adipose tissues, precluding them from circulating and thus resulting in starvation sensitivity at the organismal level [24]. We therefore tested whether Imp-L2 functions as an inhibitor of IIS under stress conditions. We exposed wild-type and *Imp-L2 *mutant early third instar larvae to various starvation conditions and scored for survival. Larvae lacking *Imp-L2 *showed a massive increase in mortality rate when exposed to 1% glucose or PBS for 24 hours (Figure 4c). To test whether the inability of the mutant larvae to cope with starvation was due to a failure in adjusting IIS, we monitored PIP_3 _levels under these conditions. Whereas control flies showed a decrease of PIP_3 _levels when exposed to complete starvation for 4 hours (Figure 4a), *Imp-L2 *mutant larvae still contained PIP_3 _levels that were comparable to those of control larvae reared on normal food (Figure 4b), suggesting that Imp-L2 is necessary to adjust IIS under starvation conditions. The fact that PIP_3 _levels were also slightly reduced in *Imp-L2 *mutants upon starvation could be attributed to the downregulation of *dilp3 *and *dilp5 *at the transcriptional level [18].

The dampening of IIS upon starvation could be achieved either by enhanced secretion of stored Imp-L2 or by an upregulation of Imp-L2 production. Indeed, expression profiling revealed a slight upregulation of *Imp-L2 *after 12 hours complete starvation [27]. We could not detect a change in Imp-L2 protein expression in the brain, the ring gland or the gut after complete starvation for 24 hours (data not shown). However, Imp-L2 was induced in fat body cells, where it appeared in vesicle-like structures (Figure 4d). Thus, under adverse nutritional conditions, *Drosophila *larvae weaken IIS by upregulating Imp-L2 expression in the fat body.

## Discussion

IIS signaling has evolved in animals to regulate growth and metabolism in accordance with environmental conditions. Appropriate IIS activity is ensured at several levels, including the controlled expression of binding partners of the extracellular ligands. Surprisingly, the well-characterized vertebrate IGFBPs have no obvious homologs in lower organisms. Here, we used a genetic strategy to search for negative regulators of IIS in *Drosophila*. Our approach led to the identification of Imp-L2 as a functional insulin-binding protein and antagonist of IIS.

*Imp-L2 *encodes a secreted peptide containing two Ig C2-like domains. Consistent with its secretion, the effects of *Imp-L2 *overexpression are non-autonomous. Tissue-specific over-expression of *Imp-L2*, for example in the larval fat body, results in a systemic response, and the entire animal is impaired in its capacity to grow. Conversely, the loss of *Imp-L2 *function produces larger animals. Our analysis of IIS activity (by means of the tGPH reporter *in vivo*) shows that Imp-L2 functions to downregulate IIS. We further show that wild-type Imp-L2 – but not a truncated version lacking the second Ig C2-like domain – binds Dilp2, consistent with previous findings that Imp-L2 binds human insulin, IGF-I, IGF-II and proinsulin [22].

Thus, despite lacking any clear ortholog of the classical IGFBPs with their characteristic amino-terminal IGFBP motifs, invertebrates such as flies can regulate IIS activity at the level of the ligands as a result of Imp-L2 expression. Orthologs of Imp-L2 are present in *C. elegans*, *Apis mellifera*, *Anopheles gambiae*, *Spodoptera frugiperda *and *Drosophila pseudoobscura*. Importantly, the second Ig C2-like domain of Imp-L2 also has sequence homology to the carboxyl terminus of IGFBP-7, which is the only IGFBP that, besides binding to IGFs, also binds insulin (although this binding could not be detected in a different assay [7]). We speculate that Imp-L2 resembles an ancestral insulin-binding protein and that IGFBP-7 evolved from such an ancestor molecule by replacing the amino-terminal Ig C2-like domain with the IGFBP motif.

Interestingly, Dilp2 and Imp-L2 are found in a complex with dALS (acid-labile subunit [28]). In vertebrates, most of the circulating IGFs are part of ternary complexes consisting of an IGF, IGFBP-3 and ALS [29]. These ternary complexes prolong the half-lives of the IGFs and restrict them to the vascular system, because the 150 kDa complexes cross the capillary barrier very poorly. IGFs can also be found in binary complexes of about 50 kDa with several IGFBP species but there is only little (< 5%) free circulating IGF [29]. Thus, it will be interesting to analyze the composition and bioactivities of Dilp2/Imp-L2/ALS complexes in *Drosophila*.

IIS coordinates nutritional status with growth and metabolism in developing *Drosophila*. It has been shown that IIS regulates the storage of nutrients in the fat body [24], an organ that resembles the mammalian liver as the principal site of stored glycogen [30]. Even under adverse nutritional conditions, fat body cells with increased IIS activity continue stockpiling nutrients, thereby limiting the amount of circulating nutrients, which induces hypersensitivity to starvation of the larva [24]. Upon starvation, the expression of *dilp3 *and *dilp5 *is suppressed at the transcriptional level in the m-NSCs [18]. Our study reveals an additional layer of IIS regulation. Whereas *Imp-L2 *is not expressed in the fat body of fed larvae, starved animals induce *Imp-L2 *expression in the fat body to systemically dampen IIS activity. A lack of this control mechanism is lethal under unfavorable nutritional conditions, as *Imp-L2 *mutant larvae fail to cope with starvation.

## Conclusion

Our study provides the first functional characterization of an insulin-binding protein in invertebrates. We have identified Imp-L2 as a secreted antagonist of IIS in *Drosophila*. Given the sequence homology of their Ig domains, we propose that Imp-L2 is a functional homolog of vertebrate IGFBP-7. Because both Imp-L2 and IGFBP-7 are potent inhibitors of growth and Imp-L2 is essential for the endurance of periods of starvation, it is likely that the original function of the insulin-binding molecules was to keep IIS in check when nutrients were scarce. Thus, in accordance with several reports suggesting that IGFBP-7 acts as a tumor suppressor, loss of IGFBP-7 may provide tumor cells with a growth advantage under conditions of local nutrient deprivation, such as in prevascularized stages of tumorigenesis.

## Materials and methods

### Fly stocks

The following fly stocks and transgenes have been used: *y w*; *w*^1118^; arm-*Gal4*; Act5C-*Gal4*; UAS-*GFP*; UAS-*lacZ *(all from the Bloomington *Drosophila *stock center); GMR-*Gal4 *(a gift of M. Freeman); ppl-*Gal4 *(a gift of M. Pankratz); UAS-*dInR *[17]; Df(3L)AC1 [17]; tGPH [24]; GMR>*w*^+^>*Gal4 *[17]; UAS-*dPTEN *[31]; UAS-*dilp2 *[17]; GE24013 (GenExel). All crosses were performed at 25°C unless stated otherwise.

### EP screen and isolation of *Imp-L2 *alleles

The EP screen that led to the identification of *Imp-L2 *will be described elsewhere (F.W., W.B., H.S., D. Nellen, K. Basler and E.H., unpublished work). A double-headed EP element (containing ten Gal4-binding sites at each end) suppressing the GMR-*Gal4*, UAS-*InR *big eye phenotype was identified in the *Imp-L2 *locus. Plasmid rescue of EP5.66 revealed that it was inserted 6,969 bp upstream of the first exon of the *Imp-L2-RB *(CG15009-RB) transcript.

To obtain loss-of-function alleles of *Imp-L2*, we performed an EMS mutagenesis screen in which we selected mutated chromosomes carrying EP5.66 that could no longer suppress the *dInR *overexpression phenotype in the eye. EP5.66 males were fed with 25 mM EMS and subsequently crossed to GMR-*Gal4*, UAS-*dInR *virgins. 39,000 F1 flies were screened for a reversion of the suppressive effect of EP5.66 on the growth phenotype caused by GMR-*Gal4*, UAS-*dInR*. Only one of the identified reversion lines, *Imp-L2*^*MG*2^, could be confirmed. Sequencing the genomic DNA of *Imp-L2*^*MG*2 ^revealed a point mutation that resulted in a truncation (Trp232Stop).

In order to generate additional *Imp-L2 *mutants, the P-element GE24013 (marked with *white*^+^) inserted 102 bp upstream of the first exon of the Imp-L2-RC transcript was mobilized by supplying Δ2–3 transposase. Jump starter males were mated with balancer females, and single F1 *w*^- ^males were recrossed to balancer virgins. Stocks (350) were established and molecularly tested for deletions by single-fly PCR using several primer pairs, leading to the identification of the alleles *Imp-L2*^*Def*42^, *Imp-L2*^*Def*20^, *Imp-L2*^*Def*35^, *Imp-L2*^*Def*223 ^and *Imp-L2*^*Def*29^.

### Construction of plasmids

In order to generate the UAS-*Imp-L2 *construct, a *Bgl*II/*XhoI *fragment of *Imp-L2 *was excised from the *Imp-L2-RB *containing cDNA clone LP06542 and inserted into pUAST [32]. To obtain UAS-*s.Imp-L2*, the second and third exons of *Imp-L2 *were amplified by PCR from genomic DNA. The fragment was subcloned into pCRII-Topo (Invitrogen). The insert was then excised with *Eco*RI and cloned into pUAST [32]. Because of the lack of the first exon of the *Imp-L2-RB *transcript (containing three upstream open reading frames), UAS-*s.Imp-L2 *has a stronger phenotype than UAS-*Imp-L2*. The EP element contains ten UAS sites, whereas the UAS transgenes contain only five.

For the generation of the genomic rescue construct, the genomic fragment L2G314 (kindly provided by J. Natzle) was used. The fragment (5 kb of genomic sequence upstream of the first exon of the *Imp-L2-RB *transcript and 1 kb downstream of the third exon) was excised with *Bam*HI and *Asp*718 and inserted into the pCaSpeR-4 transformation vector [33].

The *Flag-dilp2 *construct was created by PCR amplification of the *dilp2 *coding sequence without the signal peptide sequence from the full-length cDNA clone, EST GH11579 (obtained from Research Genetics). The resulting PCR product was then equipped with the hemagglutinin signal peptide sequence and a Flag tag and inserted into pUAST [32].

### Cell culture

*Drosophila *embryonic S2 cells were grown at 25°C in Schneider's *Drosophila *medium (Gibco/Invitrogen) supplemented with 10% heat-inactivated fetal-calf serum (FCS), penicillin and streptomycin.

For the construction of the stably expressing *Flag-dilp2 *cell line, S2 cells were co-transfected with UAS-*Flag-dilp2*, Act-*Gal4 *and a third vector containing a blasticidin-resistance gene, using effectene transfection reagent (Qiagen). Two days after the transfection, the selection medium (Schneider's containing 10% FCS and 25 μg/ml blasticidin) was added to the cells. After 10 days the selection medium was replaced by Schneider's containing 10% FCS and 10 μg/ml blasticidin.

### *In vitro *pulldown assay

S2 cells expressing *Flag-dilp2 *were grown to confluence in 175 cm^2 ^culture flasks, washed with ice-cold PBS and extracted in immunopreciptiation (IP) buffer (120 mM NaCl, 50 mM Tris pH 7.5, 20 mM NaF, 1 mM benzamidine, 1 mM EDTA, 6 mM EGTA, 15 mM Na_4_P_2_O_7_, 0.5% Nonidet P-40, 30 mM β-glycerolphosphate, 1× Complete Mini protease inhibitor (Roche)). After incubation for 15 min on an orbital shaker at 4°C, solubilized material was recovered by centrifugation at 13,000 rpm for 15 min and supernatants were collected. Anti-Flag antibody (5 μg, Sigma M2, F3165) was added and incubated over night at 4°C while rotating. Protein G sepharose beads (Amersham Biosciences) were added for 2 h and the beads were washed four times with IP buffer. Cell lysate from native S2 cells was subjected to the same procedure and the resulting beads were used as control. To verify the immunoprecipitation, a fraction of the beads was incubated with SDS loading buffer (62.5 mM Tris-HCl pH 6.8, 20 mM DTT, 2% SDS, 25% glycerol, 0.02% bromophenol blue) for 5 min at 90°C and the proteins were separated by SDS-PAGE. The presence of Flag-Dilp2 was confirmed by immunoblotting.

For the *in vitro *translation the *Imp-L2-RC *cDNA (SD23735) was cloned into pCRII.1 (Invitrogen) downstream of the SP6 polymerase promoter. As a control, the point mutation encoding a non-functional, truncated version of Imp-L2 (identified in the EMS reversion mutagenesis) was inserted into *Imp-L2-RC *(in pCRII.1 see above) using the Quick-Change site-directed mutagenesis protocol (Stratagene). Both the *Imp-L2 *and the *Imp-L2*^*MG*2 ^constructs were translated *in vitro *using the TNT Quick coupled transcription/translation system (Promega) according to the manufacturer's protocol. Briefly, 2 μg of DNA was incubated with 20 μCi [^35^S]methionine and 20 μl TNT Quick Master Mix in a total volume of 25 μl for 90 min at 30°C. The product (2.5 μl) was used in the *in vitro *pulldown assay together with Flag-Dilp2 bound to beads or with control beads in IP buffer containing 0.05% NP-40. The reaction was rotated overnight at 4°C, the beads were washed six times with IP buffer (0.05% NP-40) and incubated with SDS loading buffer containing 100 mM DTT for 10 min at 80°C. The dissociated proteins were separated using SDS-PAGE and detected by autoradiography.

### Phenotypic analyses

Freshly eclosed flies were collected, separated according to sex, placed on normal fly food for 3 days and anesthetized for 1 min with ether before weighing. Weight was determined using a Mettler Toledo MX5 microbalance. Wing size was analyzed as described [34]. ImageJ 1.32j software was used to determine the pixels of the wing area. Scanning electron microscope pictures were taken from adult flies that were critical-point dried and coated with gold.

Heat-shock induced overexpression clones (*y*, *w*, hs-*Flp*; GMR>*w*^+^>*Gal4*) were induced 24–48 h after egg-laying by a 1 h heat shock at 37°C. Tangential sections of adult eyes were generated as described [35].

### Starvation experiments

For all starvation experiments, eggs were collected for 2 h on apple agar plates supplemented with yeast. After 72 h, larvae were quickly washed in PBS and transferred either to a new apple agar plate with yeast (normal food, called 'yeast' henceforth), a solution containing 20% glucose in PBS, or a filter paper soaked with 1% glucose in PBS or PBS only. After 24 h, dead larvae were counted.

For the tGPH reporter analysis under starvation, the 'PBS' or 'yeast' conditions were used (see above). After 4 h starvation, larvae were dissected in PBS, fixed and stained with Hoechst. Pictures were taken using a Leica SP2 confocal laser scanning microscope.

### Immunohistochemistry and *in situ *hybridization

The antibody against Imp-L2 was described earlier [25] and kindly provided by J. Natzle (Department of Molecular and Cellular Biology, University of California, Davis, USA). Antibody staining against Imp-L2 was performed using the following dilutions: rat anti-Imp-L2 (1:500), donkey anti-rat-FITC (1:200, Jackson). Other antibodies used were: anti-β-galactosidase (1:2,000, polyclonal, rabbit), an antibody against the carboxyl terminus of dInR (INRcT, 1:10,000) [36]. Nuclei were either stained with 4',6-diamidino-2-phenylindole (DAPI) or Hoechst. Pictures were taken using a Leica SP2 confocal laser scanning microscope.

RNA *in situ *hybridization using digoxigenin-labeled probes was performed as described [17]. The probes against *Imp-L2 *were derived from *s.Imp-L2 *in a pBluescript SK^+ ^vector.

## Additional data files

The following file is available: Additional data file 1 contains three figures. Figure S1 shows that the overexpression of *Imp-L2* results in reduced PIP_3_ levels *in vivo*. In Figure S2, the dynamic expression pattern of Imp-L2 during development is shown. Figure S3 demonstrates that a reduction in Dilp levels enhances the growth-inhibitory effect of *Imp-L2*.

## Supplementary Material

Additional data file 1 Figure S1 shows that the over-expression of *Imp-L2 *results in reduced PIP_3 _levels *in vivo*. In Figure S2, the dynamic expression pattern of Imp-L2 during development is shown. Figure S3 demonstrates that a reduction in Dilp levels enhances the growth-inhibitory effect of *Imp-L2*. Click here for file
