# Supplementary material for: Imp-L2, a putative homolog of vertebrate IGF-binding protein 7, counteracts insulin signaling in Drosophila and is essential for starvation resistance
Source: J Biol. 2008 Apr 15;7(3):10. doi: 10.1186/jbiol72 (PMC2323038; doi:10.1186/jbiol72)
Supplement: Additional data file 1 — Figure S1 shows that the over-expression of Imp-L2 results in reduced PIP3 levels in vivo. In Figure S2, the dynamic expression pattern of Imp-L2 during development is shown. Figure S3 demonstrates that a reduction in Dilp levels enhances the growth-inhibitory effect of Imp-L2. [file jbiol72-S1.pdf]

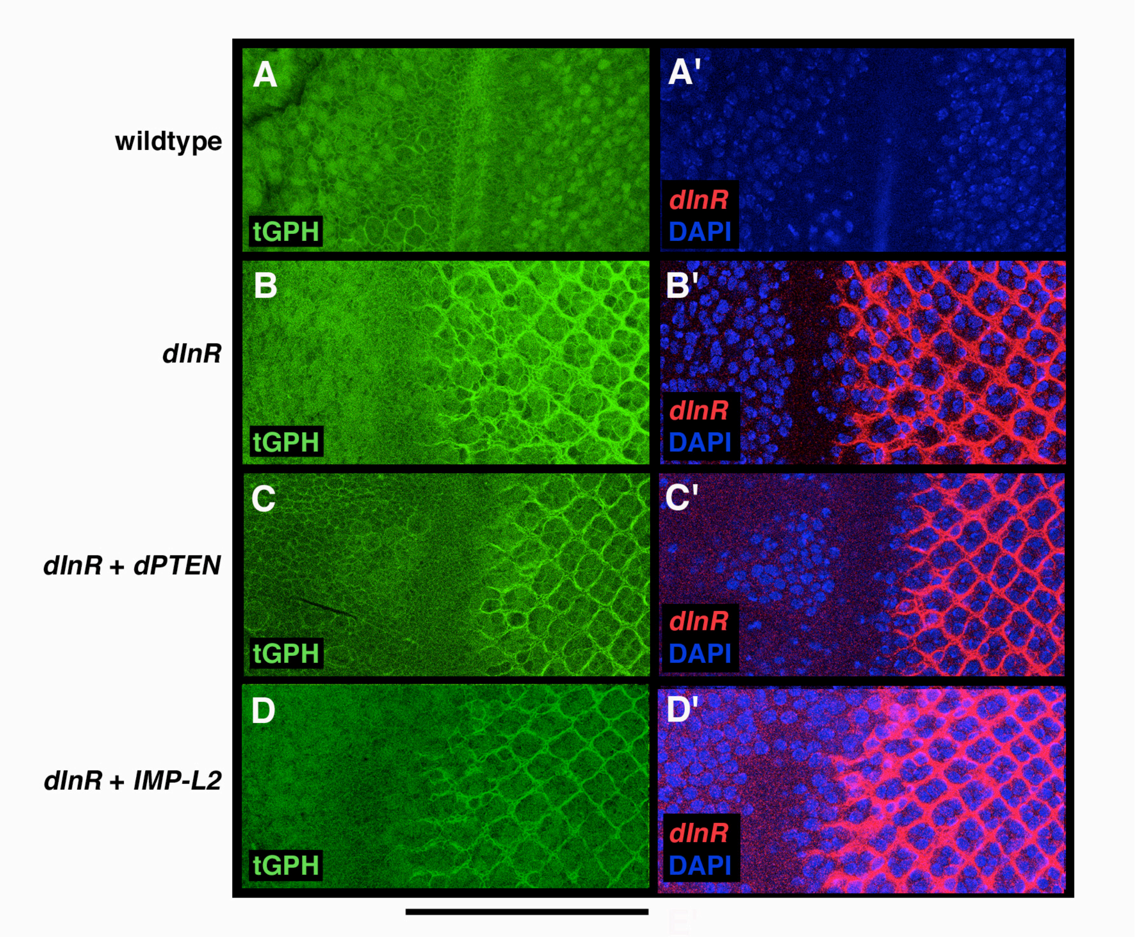

Figure S1 ***Imp-L2* reduces PIP$_3$ levels**

(A-D) Localization of the tGPH reporter (green) in eye imaginal discs of third instar larvae. (A'-D') Expression of dInR protein is shown in red, and nuclear DNA staining (DAPI) in blue. Posterior is always to the right. (A) In wild-type eye imaginal discs, IIS is low as reflected by the basal tGPH reporter recruitment to the membrane. (B) Overexpression of *dInR* posterior to the morphogenetic furrow causes a strong membrane relocation of tGPH. (C) Simultaneous overexpression of *dInR* and *dPTEN* reduces membrane-bound tGPH partially. (D) Like *dPTEN*, *Imp-L2* overexpression reduces the *dInR*-induced membrane localization of tGPH. Note that dInR protein levels are unaffected (B'-D'). The region posterior to the morphogenetic furrow is indicated by the bar below (D). Genotypes: (A, A') *y, w*; *tGPH*/+, (B, B') *y, w*; UAS-*dInR*, *tGPH*/+; GMR-*Gal4*/+, (C, C') *y, w*; UAS-*dInR*, *tGPH*/+; GMR-*Gal4*/UAS-*dPTEN*, (D, D') *y, w*; UAS-*dInR*, *tGPH*/+; GMR-*Gal4*/UAS-*s.Imp-L2*.

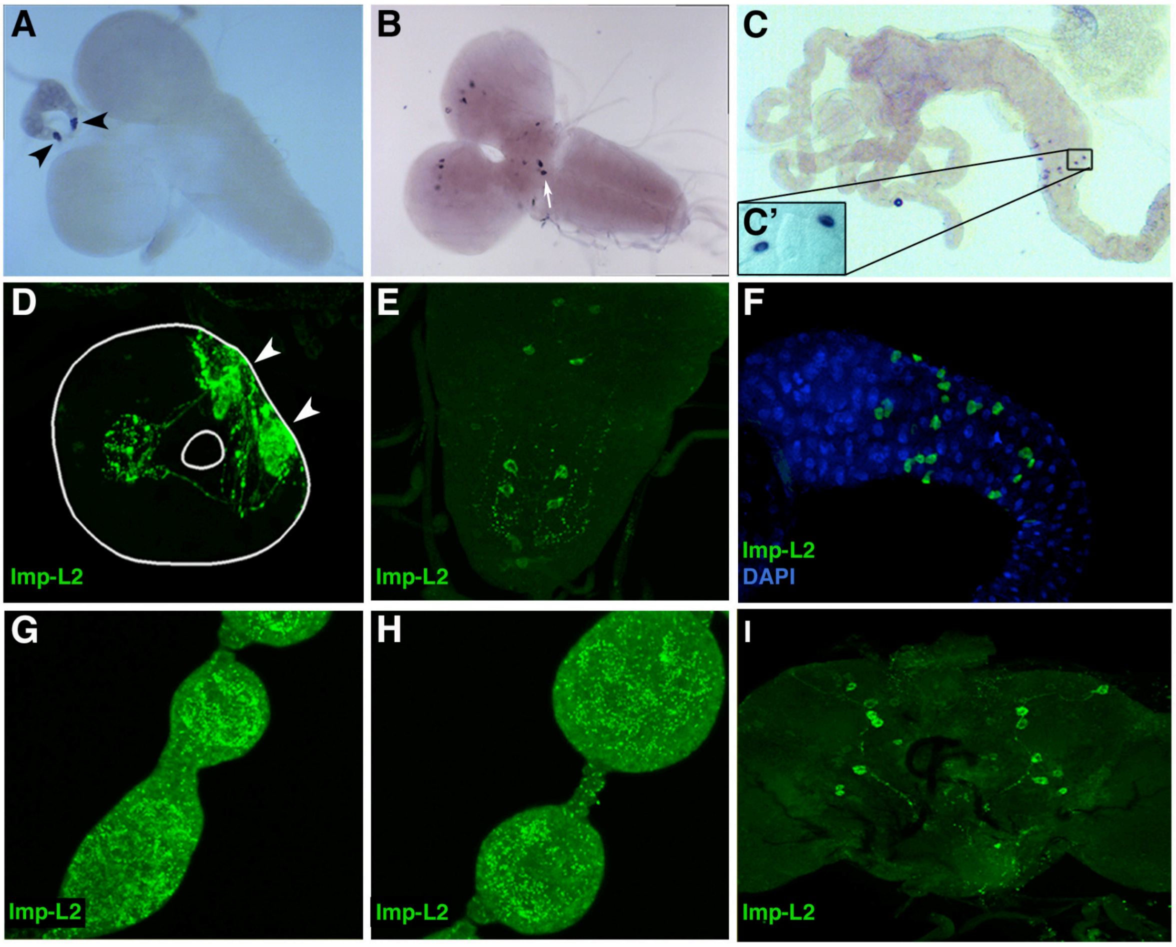

Figure S2 ***Imp-L2* is expressed in a specific pattern throughout development**

(A) *In situ* hybridization with *Imp-L2* antisense probes in third instar larvae strongly stains cells of the CC (black arrowheads). (B) Prolonged detection also reveals staining in several neurons of both brain hemispheres and the SOG region (white arrow). C) A cluster of cells with unknown function in the anterior midgut epithelium expresses *Imp-L2*. Note that these cells are much smaller than the surrounding cells of the epithelium (C' is a higher magnification of C). (D-I) Antibody (Ab) staining of larval and adult tissues with an Imp-L2-Ab (green). (D) The CC (white arrowheads) express Imp-L2 protein at high levels. The corpora allata (CA) are innervated by Imp-L2 expressing axons. (E) Eight neurons in the ventral ganglion, which resemble *dilp7* expressing cells, also express Imp-L2 protein. (F) Larval midgut cells express Imp-L2 protein at high levels. (G-H) In the ovaries of adult females, Imp-L2 is expressed in vesicle-like structures in early stages of oogenesis. During later stages of oogenesis, the Imp-L2 containing vesicles disappear (H). (I) Like in larvae, Imp-L2 is expressed in distinct neurons of both brain hemispheres of adult males and females.

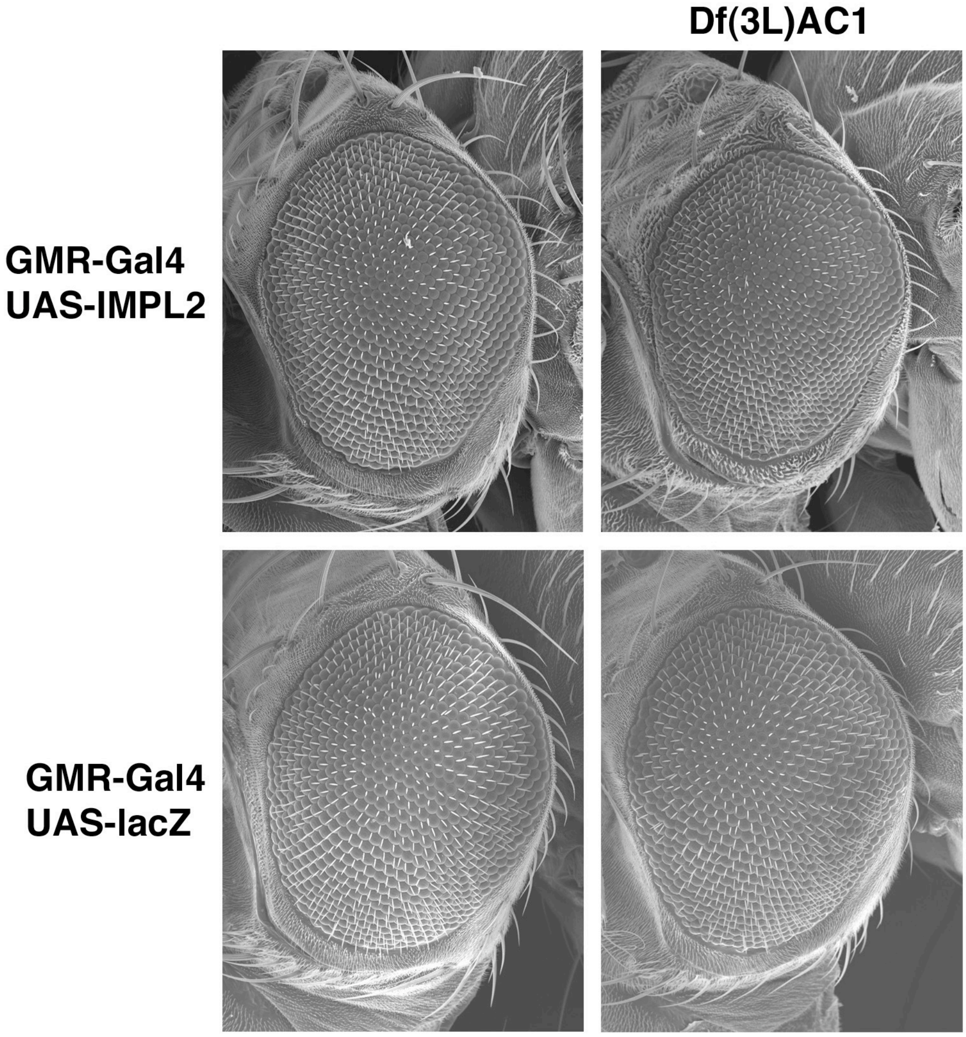

Figure S3 A reduction in Dilp levels dominantly enhances the Imp-L2-induced reduction in eye size

SEM pictures of female eyes. The deficiency *Df(3L)AC1*, which uncovers a region containing *dilp1* to *5*, already results in a slight size decrease (compare bottom left to bottom right). However, in a GMR-*Gal4*, UAS-*Imp-L2* background, the dominant size reduction of *Df(3L)AC1* is significantly enhanced (compare the size reduction between top left to right and bottom left to right).
